# Sun Exposure Behaviors and Knowledge Among the At‐Risk Population: Results From an International Survey, the HELIOS Project

**DOI:** 10.1111/phpp.13014

**Published:** 2024-11-11

**Authors:** Thierry Passeron, Brigitte Dreno, Susana Puig, Chee Leok Goh, Hee Young Kang, Fatimata Ly, Akimichi Morita, Jorge Ocampo Candiani, Sergio Schalka, Liu Wei, Anne‐Laure Demessant‐Flavigny, Caroline Le Floc’h, Delphine Kerob, Henry W Lim, Jean Krutmann

**Affiliations:** ^1^ Department of Dermatology Côte d'Azur University, Nice University Hospital Center Nice France; ^2^ INSERM U1065, C3M Côte d'Azur University Nice France; ^3^ Nantes University Univ Angers, INSERM, Immunology and New Concepts in ImmunoTherapy, INCIT Nantes France; ^4^ Melanoma Unit, Dermatology Department Barcelona University Hospital Clinic Barcelona Spain; ^5^ National Skin Centre Singapore Singapore; ^6^ Department of Dermatology Ajou University School of Medicine Suwon South Korea; ^7^ Department of Dermatology Cheikh Anta Diop Dakar University, EPS Institute of Social Hygiene Dakar Senegal; ^8^ Department of Geriatric and Environmental Dermatology Nagoya City University Graduate School of Medical Sciences Nagoya Japan; ^9^ Department of Dermatology Medical Faculty University Hospital of Nuevo Leon Monterrey Mexico; ^10^ Medcin Skin Research Center and Biochemistry Department Chemistry Institute of Sao Paulo University Sao Paulo Brazil; ^11^ Department of Dermatology The General Hospital of air Force PLA Beijing China; ^12^ La Roche‐Posay International Levallois‐Perret France; ^13^ Department of Dermatology Henry Ford Health Detroit Michigan USA; ^14^ IUF Leibniz Research Institute for Environmental Medicine Dusseldorf Germany; ^15^ Medical Faculty Heinrich‐Heine‐University Dusseldorf Germany

**Keywords:** epidemiology, photoprotection, risk assessment, sunburn/prevention and control, sunlight adverse effects, survey

Large‐scale prevention campaigns have significantly enhanced public awareness of the risks of excessive exposure to sunlight and its association with skin cancers. However, changing behavior remains a significant challenge for the general population [1] particularly in vulnerable groups with a personal history of skin cancer [2] or medication‐induced sun sensitivity [3]. In organ transplantation recipients (OTRs), over half develop skin cancer due to long‐term immunosuppressive therapy and excessive UV radiation [4].

A recent study by Daniela L. Domínguez Bueso et al. [5] concluded that a significant proportion of skin cancer survivors in the US still do not engage in frequent sun protection behavior. We aim to expand this perspective globally and evaluate the behaviors, attitudes toward sun exposure and knowledge among individuals at increased risk of sunlight‐related damage.

The Helios project is based on data from an extensive survey conducted from 28 September to 18 October 2021 using an Ipsos Panel with 17,001 participants from 17 countries spanning five continents [6]. This subanalysis focused on two primary “at‐risk” population (*N* = 2114): those with a history of skin cancer or immunosuppressant use—for their condition or after an organ transplant—referred to as the skin cancer/ImmunoSuppressant (SK/IS) subpopulation (*n* = 1731, of whom 434 OTRs) and those with a history of photodermatoses or photosensitive treatments—the P/PT subpopulation (*n* = 1047).

Statistical analyses were based on frequency tables, means, standard deviations, 95% confidence intervals, and two‐sided chi‐square tests with a 0.05 significance level to compare different subgroups [6].

The at‐risk population represented 12% (*n* = 2114) of the overall population (*N* = 17,001), including SK/IS subpopulation (10%, *n* = 1731) and the P/PT subpopulation (6%, *n* = 1047) (Tables 1 and 2).

**TABLE 1:** Characteristics of the at‐risk population.

Item	All (*N* = 17,001)	At‐risk (*N* = 2114)	*p*
Gender, male	49%	52%	0.001
Age, average (SD), years	44.5 (16.3)	48 (16.9)	< 0.001
Phototypes			
I–II	47%	56%	< 0.001
III–IV	43%	38%	< 0.001
V–VI	10%	6%	< 0.001
Do you have your moles checked by a dermatologist?			
At least once a year (ST)	16%	49%	< 0.001
Perception of skin tan and associated risks			
Would you say, … (ST, yes)			
Exposure to the sun can cause skin health problems	88%	90%	0.001
Exposure to the sun accelerates skin aging	81%	87%	< 0.001
You cannot imagine coming back from holidays without being tanned	49%	59%	< 0.001
A tan makes a person look healthy	64%	72%	< 0.001
Value of photoprotection			
According to you, is the risk of developing skin cancer very much, a little, not really or not at all linked to…?			
A lack of protection during exposure to the sun	76%	83%	< 0.001
Chronic exposure to the sun	74%	82%	< 0.001
History of severe sunburn during childhood or adolescence	62%	77%	< 0.001
How much do you regret not having better protected yourself from the sun in the past? (Yes, ST)	57%	79%	< 0.001
Photoprotection measure			
In general, you protect yourself from the sun …	23%	40%	< 0.001
All year round, whatever the season (ST)			
When you are exposed to the sun, do you use			
All practices^a^ systematically or often (ST)	12%	27%	< 0.001
When you are exposed to the sun, how often do you apply your sunscreen?			
Base (individuals using sunscreen, even rarely)	*n* = 13,434	*n* = 1903	
Every two hours or More often (after each bath/after sweating) (ST)	26%	26%	—
When your skin is getting tanned, do you keep applying your sunscreen?			
With a reduced frequency or protection factor (ST)	44%	38%	< 0.001
What is the level of UVB protection of the sunscreen you are using most often? *High or very high* (ST)	65%	66%	—
Value of photoprotection			
According to you, is the risk of developing skin cancer very much, a little, not really or not at all linked to…?			
History of severe sunburn during childhood or adolescence	62%	77%	< 0.001
A lack of protection during exposure to the sun	76%	83%	< 0.001
Chronic exposure to the sun	74%	82%	< 0.001
How much do you regret not having better protected yourself from the sun in the past? (Yes, ST)	57%	79%	< 0.001
Knowledge of sunrays			
How well, if at all, do you feel you understand: (Well, ST)			
The differences between UVB and UVA	30%	46%	< 0.001
SFP	46%	62%	< 0.001
UVA index	32%	50%	< 0.001
Visible light	48%	62%	< 0.001
IR light	51%	61%	< 0.001
Knows at least one element	75%	88%	< 0.001

Abbreviations: DK, do not know; ISD, immunosuppressive drugs; OTR, organ transplant recipient; PSD, photosensitive drugs; SD, standard deviation; ST, subtotal; UVA (UVB), ultraviolet A (B) sun rays.

^a^ All practices: wearing protecting clothing, hat/cap and sunglasses, seeking shade, refraining from sun exposure during the peak UV index, applying sunscreen.

**TABLE 2:** Characteristics of the at‐risk subpopulations SK/IS and P/PT^a^.

Item	SK/IS (*n* = 1731)	P/PT (*n* = 1047)	*p*
Gender, male	53%	54%	
Age, average (SD), years	49 (17.1)	42.2 (15.4)	< 0.001
Phototypes			
I–II	57%	54%	—
III–IV	37%	38%	—
V–VI	6%	8%	0.04
Do you have your moles checked by a dermatologist?			
At least once a year (ST)	51%	51%	—
Perception of skin tan and associated risks			
Would you say, … (ST, yes)			
Exposure to the sun can cause skin health problems	90%	87%	0.001
Exposure to the sun accelerates skin aging	88%	83%	< 0.001
You cannot imagine coming back from holidays without being tanned	59%	71%	< 0.001
A tan makes a person look healthy	73%	75%	—
Value of photoprotection			
According to you, is the risk of developing skin cancer very much, a little, not really or not at all linked to…?			
A lack of protection during exposure to the sun	84%	81%	0.04
Chronic exposure to the sun	82%	79%	0.05
History of severe sunburn during childhood or adolescence	78%	75%	—
How much do you regret not having better protected yourself from the sun in the past? (Yes, ST)	80%	83%	0.05
Photoprotection measure			
In general, you protect yourself from the sun … All year round, whatever the season (ST)	41%	25%	< 0.001
When you are exposed to the sun, do you use	28%	35%	< 0.001
All practices^b^ systematically or often (ST)			
When you are exposed to the sun, how often do you apply your sunscreen?			
Base (individuals using sunscreen, even rarely)	*n* = 1563	*n* = 949	
Every 2 h or More often (after each bath/after sweating) (ST)	26%	22%	0.02
When your skin is getting tanned, do you keep applying your sunscreen?			
With a reduced frequency or protection factor (ST)	38%	38%	—
What is the level of UVB protection of the sunscreen you are using most often? High or very high (ST)	66%	60%	< 0.001
Value of photoprotection			
According to you, is the risk of developing skin cancer very much, a little, not really or not at all linked to…?			
History of severe sunburn during childhood or adolescence A lack of protection during exposure to the sun Chronic exposure to the sun	78%	75%	—
	84%	81%	0.04
	82%	79%	0.05
How much do you regret not having better protected yourself from the sun in the past? (Yes, ST)	80%	83%	0.05
Knowledge of sunrays			
How well, if at all, do you feel you understand: (Well, ST)			
The differences between UVB and UVA SFP UVA index Visible light IR light	46%	56%	< 0.001
	63%	68%	0.001
	52%	58%	< 0.001
	61%	70%	< 0.001
	61%	70%	< 0.001

Abbreviations: DK, do not know; ISD, immunosuppressive drugs; OTR, organ transplant recipient; PSD, photosensitive drugs; SD, standard deviation; ST, subtotal; UVA (UVB), ultraviolet A (B) sun rays.

^a^ The SK/IS subpopulation is made of individuals with a history of skin cancer or precancerous lesions or treated with immunosuppressive drugs for their disease or following an organ transplantation (OTR). The P/PT subpopulation is made of individuals with a history of photodermatosis or treated with photosensitive drugs.

^b^ All practices: wearing protecting clothing, hat/cap and sunglasses, seeking shade, refraining from sun exposure during the peak UV index, applying sunscreen.

The at‐risk population adopted safer protection measures than the overall population. In particular, 40% declared they used sun protection throughout the year in contrast to 23% of the total population. This was particularly true in the SK/IS population compared with the P/PT population (41% vs. 25%, respectively). Moreover, 27% of at‐risk individuals reported using all protective measures systematically or often, compared with 12% in the total population. Once again, a difference was observed between the two subpopulations: 28% of the SK/IS population and 35% of the P/PT population reported using the full range of protective measures systematically or often.

In terms of medical surveillance, 49% of the at‐risk population (51% for both SK/IS and P/PT) had their moles checked at least once a year by a dermatologist, which is much higher than in the general population (16%).

Overall, the at‐risk population was more likely to acknowledge the connection between ineffective photoprotection behaviors and the risk of developing skin cancer, such as the lack of protection during sun exposure (83% vs. 76%), chronic exposure (82% vs. 74%) and severe sunburns during childhood or adolescence (77% vs. 62%). This population also expressed more regret about their poor level of sun protection in the past than the total population (79% vs. 57% of the general population).

In the SK/IS and P/PT subpopulations, tanning and holiday activities were often combined, with 59% and 71%, respectively stating that it was inconceivable to return from holiday without a tan.

While the majority of the at‐risk population had some knowledge about at least one aspect of sunlight (88% among at‐risk vs. 75% of the total population), many misunderstandings still remain. In comparison to the total population, where 30% had some understanding of the difference between UVA and UVB, a higher yet still minority portion of the at‐risk population (46%) was familiar with the difference. This trend was mirrored in other sunlight‐related notions: 62% and 50% of at‐risk individuals understood SFP and UVA indexes, respectively (versus 46% and 32% of the total population, respectively).

The populations studied, who are at an increased risk of sunlight‐related skin damage, including skin cancers, exhibited suboptimal adherence to photoprotection measures given their health status. Although it exceeds that of the general population, photoprotection levels remain relatively low among these at‐risk populations. However, individuals at‐risk were in a position to receive superior dermatological advice.

In line with the conclusions drawn by Daniela L. Domínguez Bueso et al. [5] in a US population, the results of this international survey suggest both the overall at‐risk population and the SK/IS subpopulation including individuals with a history of skin cancer, exhibit safer attitudes towards sunlight exposure, except in terms of the frequency of sunscreen application. However, the frequent application of sunscreen item was assessed differently in both studies, which may lead to varying interpretations by the participants. Additionally, it is worth acknowledging the potential biases inherent in declarative surveys, such as cognitive and memory biases, as well as social desirability bias, which may affect both studies.

Prevailing misconceptions suggest that at‐risk populations often underestimate their individual risks in favor of the immediate psychological benefits of intentional tanning. It is noteworthy that this attitude was particularly prevalent among at‐risk individuals and has been reported even among those with a personal history of melanoma, for whom the immediate psychological benefit of tanning outweighs the long‐term consequences of exposure to sunlight [7]. This highlights the critical role of dermatologists and general practitioners in providing comprehensive education and guidance [8].

This framework should empower individuals to make decisive changes to their lifestyle and attitude towards sun protection. Special attention is needed for individuals with heightened vulnerability due to personal medical histories (including skin cancer, precancerous lesions, photodermatosis) or medical conditions (past or present use of immunosuppressive, including OTR or photosensitive medications). This attention is crucial not only for primary prevention but also throughout their medical surveillance and skin cancer screening.

## Author Contributions

All authors made substantial contributions to the conception and design and were involved in drafting the manuscript or revising it critically for important intellectual content and gave final approval.

## Ethics Statement

Ipsos is an independent Market Research Company. The survey has been carried out according to the ICC/ESOMAR code of conduct.

## Conflicts of Interest

Dr. Thierry Passeron reports personal fees from La Roche Posay during the conduct of the study; personal fees from L'Oréal, personal fees from SVR, personal fees from Symrise, personal fees from Isis Pharma, personal fees from Bioderma, personal fees from Beiersdorf, personal fees from ISDIN, personal fees from Pierre Fabre, personal fees from Hyphen, outside the submitted work. Dr. H.W. Lim is an investigator for Incyte, L'Oreal, Pfizer, and PCORI, has served as a consultant for ISDIN, Ferndale, L'Oreal, Eli Lilly and Beiersdorf, and has been a speaker on general educational session for La Roche‐Posay, Pierre Fabre, Cantabria labs, NAOS, Uriage, and Pfizer. Dr. C.L. Goh reports other support from La Roche Posay during the conduct of the study. Dr. H.Y. Kang reports personal fees from L'Oréal during the conduct of the study. Dr. F. Ly has nothing to disclose. Dr. A. Morita reports personal fees from L'Oréal during the conduct of the study. Dr.J. Ocampo Candiani reports personal fees from La Roche Posay during the conduct of the study and personal fees from Pierre Fabre outside the submitted work. Dr. S. Puig reports personal fees from La Roche Posay, during the conduct of the study; grants, personal fees and nonfinancial support from Almirall, personal fees from Amgen, personal fees and nonfinancial support from Avene, personal fees and nonfinancial support from BMS, grants and personal fees from Cantabria, grants, personal fees and nonfinancial support from ISDIN, grants from MSD, nonfinancial support from Lilly, nonfinancial support from Abbvie, personal fees and nonfinancial support from Pierre Fabre, grants, personal fees and nonfinancial support from La Roche Posay, personal fees from Pfizer, personal fees and nonfinancial support from Sanofi, grants, personal fees and nonfinancial support from Sunpharma, grants and personal fees from Roche, outside the submitted work. Dr. S. Schalka has served as an investigator for NAOS/Bioderma, Johnson& Johnson, Mantecorp Skincare Brasil and FQM Brasil. He has also served as a consultant for Pierre Fabre, ISDIN, FQM Brasil, Mantecorp Skincare Brasil, Vichy, La Roche Posay, and NAOS/Bioderma and has participated as a speaker in an educational session for Pierre Fabre, La Roche‐Posay, Eucerin, and NAOS/Bioderma. Dr. L. Wei reports personal fees from La Roche Posay during the conduct of the study. A.‐L. Demessant‐Flavigny, C. Le Floc'h, D. Le Kerob are employees of La Roche Posay. Dr. Brigitte Dreno reports personal fees from La Roche Posay International during the conduct of the study. Dr. J. Krutmann reports personal fees from La Roche Posay during the conduct of the study; grants and personal fees from Amway, grants and personal fees from Beiersdorf, grants and personal fees from bitop, grants and personal fees from Blue Lagoon, grants and personal fees from Estee Lauder, grants and personal fees from Evonik, grants and personal fees from Galderma, grants and personal fees from Henkel, grants and personal fees from Horphag, grants and personal fees from ISDIN, grants and personal fees from Kiessling, grants and personal fees from Lancaster‐Coty, grants and personal fees from La Roche Posay, grants and personal fees from L'Oréal, grants and personal fees from Lycored, grants and personal fees from Mary Kay, grants and personal fees from Mibelle, grants and personal fees from Procter & Gamble, grants and personal fees from Repairogen, grants and personal fees from RepliCel, grants and personal fees from Skinceuticals, grants and personal fees from SkinMedica, an Allergan Company, grants and personal fees from Stada, grants and personal fees from Symrise, grants and personal fees from Unilever, grants and personal fees from Vichy, grants and personal fees from Walgreen‐Boots‐ Alliance, outside the submitted work.

## Data Availability

The data that support the findings of this study are available from the corresponding author upon reasonable request.
